# A Scoping Review of Educator Proficiency Interventions in Pharmacy Education Illustrated by an Interdisciplinary Model Integrating Pedagogical Theories into Practice

**DOI:** 10.3390/pharmacy11060172

**Published:** 2023-10-30

**Authors:** Gabriella Nagy, Ferenc Arató, István G. Télessy, Aranka Varga, András Fittler

**Affiliations:** 1 Department of Languages for Biomedical Purposes and Communication, University of Pécs Medical School, 7624 Pécs, Hungary; 2 Doctoral School of Health Sciences, University of Pécs, 7624 Pécs, Hungary; 3 Department of Education and Educational Theory, Faculty of Humanities and Social Sciences, University of Pécs, 7624 Pécs, Hungary; 4 Department of Pharmaceutics, Faculty of Pharmacy, University of Pécs, 7624 Pécs, Hungary; 5 Department of Roma Studies and Educational Sociology, Faculty of Humanities and Social Sciences, University of Pécs, 7624 Pécs, Hungary

**Keywords:** pharmacy education, educator proficiency, faculty development, hidden curriculum, interdisciplinary cooperation, pedagogy

## Abstract

Pharmacy schools recognize the need for flexibility and comprehensive curricular transformation with a competency-based focus to effectively prepare for the evolving practitioner competencies and challenges of the pharmacy profession. The curricular implementation of evidence-based teaching and learning theories and practices demands educator proficiency through skills development with indispensable faculty leadership support. Our scoping review of online databases and pharmacy education-related journals aims to identify faculty development interventions or teaching proficiency programs that integrate educational and pedagogical theories. Original studies and reviews published between 2010 and 2022 were screened based on four inclusion criteria. Thirty-four manuscripts were eligible for full-text analysis, of which seven results referenced target faculty pedagogy knowledge development. Nine key messages, as Results Statements, synthesize and provide a framework for our results analysis. An ongoing Hungarian intervention model of comprehensive faculty development with strong interdisciplinary cooperation is discussed in our study to illustrate the applicability of the Results Statements through each stage of the process. Educator motivation and relatedness to students or awareness of the educator roles are intrinsic factors, which may not be easily detectable yet significantly impact teaching proficiency and student learning outcomes. The integration of evidence-based pedagogical knowledge and training in educator proficiency development contributes to the sustainability and cost-effectiveness of faculty interventions.

## 1. Introduction

Modern pharmacy education prepares professionals for a constantly changing and market-driven healthcare environment [1], which demands a flexible response from higher education institutions. Pharmacy schools recognize the need for a comprehensive curricular transformation to follow new evidence-based learning and teaching practices, to develop problem-solving skills and integrate practice-oriented pedagogical strategies [2,3,4]. Today’s “new normal” regarding teaching methods makes ample use of digital platforms, virtual simulation or online teaching–learning environments. However, the mere use of modern technology without a clear pedagogical objective does not guarantee meeting the expected learning outcomes [5].

Complex educational models formerly developed in the past decades relied on a competency-based curricular approach, which likely included problem-based or inquiry-based learning theories [6], increased interactive learning strategies [7] or integrated the use of cooperative learning [8]. New Health Sciences frameworks have also been classified to aid stakeholder decisions; however, the study authors stipulate there is no best model that meets the varying needs [9]. Stein and colleagues proposed a direct relationship between teacher skills and student academic performance [10], which has been resonating through guidelines; the Accreditation Council for Pharmacy Education (ACPE) Consensus Conference in 2013 noted most academic faculty members are not trained as teachers [11]. Strang and Baia identified the rapidly growing popularity of a Master’s program among health profession educators (MSHPEds) [11], in which the authors explicitly demonstrate a demand for pedagogical knowledge, skills and teaching proficiency development. The International Pharmaceutical Federation (FIP) Pharmaceutical Workforce Development Goals also highlight the importance of an updated, competency-based and needs-based pharmacy education embedded in a collaborative environment in which students can actively participate in their educational process [2,12]. Academic educators are responsible for creating such learning environments with the use of relevant skills and pedagogical knowledge, preferably attained through faculty-organized or interprofessional cooperation [13]. Numerous papers describe, classify and/or assess the implementation of individually developed teaching and learning curriculum programs at different schools of pharmacy [11]; however, a comprehensive review of the disseminated teaching proficiency interventions that integrates a pedagogical focus has not yet been published.

The one-tier MSc Pharmacy Program introduced in 2000 at the Faculty of Pharmacy of the University of Pécs, Hungary, is offered in two languages and has approximately 400 students enrolled. Current curricular reforms were prompted by the recognition of unresolved and unfavorable student retention rates during the 5-year training program. In response, a faculty educational research group conducted a national survey focusing on young pharmacist practitioner retrospective views, which detected an obvious need for curricular reform (“what to teach”) [2]. However, the faculty also recognized the need to improve teaching proficiency (“how to teach”). To address this need, an interdisciplinary educator development program was launched that integrated pedagogy experts to provide evidence-based science, which ensured the reliability and effectiveness of the intervention.

Aligned with the Faculty’s current interdisciplinary intervention, the authors have hypothesized that finding comprehensive faculty interventions that integrate aspects of adult teaching and learning pedagogy will effectively engage faculty staff who are generally academic staff originating from various health and natural sciences disciplines without formal training in undergraduate education. Our main goal was to identify comprehensive faculty interventions that implement evidence-based strategies for the integration of educational science into systems development.

## 2. Aims

Our scoping review aimed to identify faculty development interventions in undergraduate pharmacy education that integrate educational and pedagogical theories to support teaching proficiency programs. The timely revision provides scientific support for the ongoing comprehensive curricular reforms at the Faculty of Pharmacy of the University of Pécs, Hungary. Our study illustrates the key findings of our review and their correspondence with the current development process at our institution.

## 3. Methods

Our methodology is a scoping review initiated with ideation to select methodology in support of our research target. Based on initial title search efforts, word clouds were created for search words to define pharmacy faculty staff and pedagogy-integrated interventions depicted in existing literature. We adopted a five-step scoping review approach [14] including the following: (1) identification of the research question; (2) identification of the relevant studies; (3) selection of the studies; (4) charting relevant data; and (5) collection, summary and report of the results.

### 3.1. Identification of the Research Question

Our scoping review focused on the following question: are there reports of faculty interventions resembling our current educator development program, in which pedagogy is valued as an important contributing factor for effective faculty development and teaching proficiency interventions?

### 3.2. Identification of the Relevant Studies

Based on word cloud and initial title search results and a modified (PIO) version of the PICO (population, intervention, comparison and outcomes) framework [11], search terms were identified, including “academic pharmacy staff”, “educator proficiency” or “faculty development”. Based on the first finds, more refined terms were added to trace the intervention or outcome, including “pedagogical training”, “active-learning”, “student engagement” and “staff competency”. Medical Subject Headings (MeSHs) terms were used to refine the scope and exclude studies of other health professions (“nurs”, “medic”). Marginal terms reflecting a curricular focus (“course content”) or student internship experiences (“practitioner”) were excluded from the records. A literature search and identification of the studies was conducted from May through July 2022 in PubMed, Science Direct databases and other scientific journals that focus on education. The identification phase included target searches in education-oriented journals, such as the American Journal of Pharmaceutical Education (AJPE) and Currents in Pharmacy Teaching and Learning to trace faculty teaching development programs. Additional records were also identified through manual searches and lists of references. The search was limited to English-language publications.

### 3.3. Selection of the Studies

Prime selection criteria included relevance and recency; thus, we next excluded publications prior to 2010 to identify potential longitudinal studies and ensure the relevance of data. The publication titles were filtered in the screening phase to locate pharmacy staff teaching proficiency focusing on undergraduate education. To ensure reliability, two authors (GN and AF) independently reviewed the titles and abstracts and reached a consensus regarding the inclusion criteria, which determined eligibility. Selected articles were included: if the article’s focus was on faculty development that targeted or included pharmacy staff participants; and if the interventions integrated or used a pedagogy tool or approach.

Literature screening revealed that reviews and more conceptual studies may not focus only on faculty educators or specify interventions, yet are likely to disseminate relevant data on the application of pedagogy theories for curricular development in pharmacy education. Therefore, reviews and conceptual studies were also included: if they presented detailed references to pharmacy educator pedagogy; or if they presented curricular development plans referencing the use of pedagogy and the interventions impacted undergraduate student benefits. Non-peer-reviewed and non-full-text articles were excluded to ensure the reliability of data. Non-English language articles and book formats were also excluded.

### 3.4. Charting of the Data

We selected the records based on the inclusion criteria, and extracted data were inserted into an Excel spreadsheet to ensure consistency in the analysis. Data extraction by the first author (GN) was followed by the critical review of the pharmacy educator author (AF). Peer author revision was useful since we found a matching publication focus with the relevant criteria most challenging due to the diversity of topics and the relatively large number (n = 34) of selected articles. The range of topics included a search for the definition of teaching excellence, perceptions of a faculty peer observation program, the perceived impact of a faculty development committee, faculty perceptions on introducing team-based learning across a pharmacy curriculum, how to conduct education-related research and interfaculty collaboration to improve educator proficiency (without referencing educator pedagogy).

Demographic and thematic categories for classification and analysis were also determined by the two authors. Demographic data included the year and location of publication, while thematic categories included the target population/study focus, intervention characteristics, assessment of the results, beneficiaries of the intervention and educational expert cooperation.

### 3.5. Collection, Summary and Report of the Results

The collection of the data required an in-depth review and critical evaluation of the selected (n = 34) full-text publications in the Excel spreadsheet during the eligibility phase (see Table S1). The critical evaluation involved decision-making of whether to include or exclude each record from the results. Our decisions were based on the inclusion criteria in Section 3.3.

The five core thematic categories for data classification and analysis were applied as follows. The target population described active intervention participants or the study focus. Intervention characteristics (mentorship program, course, workshop) included the identification and details of specific pedagogy. Intervention results included reports of the objective assessment (participant evaluations, student test scores), subjective measures (participant survey, student feedback) or absence of an evaluation. The beneficiary category clarified the direct or indirect intervention impact on student pharmacists to exclude non-educational settings. We also recorded interdisciplinary support through educational expert cooperation. The additional category for key findings or take-home messages provided synthesis or pedagogical aspects of the interventions potentially valuable for a large audience (see Table 1).

Multiple online meetings provided a platform for discussions to reach consensus on data analysis and data supervision. The two authors (GN, AF) independently reviewed the records and if differences sprang up mostly from the identification of the publication focus, discussion helped to reach a consensus. To ensure validity, the two-stage eligibility process involved two educational expert authors, FA and IT, who individually evaluated the data sheet. Full consensus was reached to establish the results. Consultation with the educational experts regarding the evaluation of pedagogical content contributed professional expertise and ensured accurate results. For example, a selected study with objective measures of team-based learning outcomes without referencing educator skills development was recommended for exclusion [15].

**Table 1:** Summary of publications included in the scoping review of pedagogy development concepts in pharmacy education.

Author, Date, Country and Publication Category	Participants (Population)	Pedagogy Method Used or Referenced (Intervention)	Evaluation of the Results (Outcome)	Interdisciplinary Cooperation	Key Findings
**Johnson MS et al. (2013) USA, study** [16]	Participants were pharmacy residents, new faculty, residency preceptors; new program component introduced formal pedagogy seminars (prior: only monthly discussions) with educational expert cooperation providing foundational pedagogy knowledge	Pedagogy seminars (12 lectures), didactic components required two participant lectures, small group facilitation, experiential teaching, development of teaching philosophy statement and teaching portfolio	Participant subjective evaluations included pre-and post-intervention feedback on program benefits, formal training and experiential performance; formal objective evaluation of participant experiential performance was given by mentors based on objective pedagogy assessment factors	Yes (in pedagogy seminars)	Validity and relevance of pedagogical knowledge was ensured by educational experts in the design, scheduling and facilitation of didactic seminars. A joint school of pharmacy and school of education program has increased resident-perceived teaching abilities and confidence.
**Edwards RA et al. (2014) USA, study** [1]	Motivate pharm. educators in improving teaching methodology, practical development through sharing peer practical teaching experience	A “new-to-you” teaching method shared, no staff education on pedagogy in faculty intervention	Self-evaluative qualitative feedback survey	No	A teaching challenge motivated most of the faculty members to try something new. Links between evidence-based principles and day-to-day activities were strengthened by the peer-teaching method.
**Mukhalalati BA et al. (2019) Qatar, review** [17]	Literature review using the term “andragogy”; to synthesize key learning theories applicable in the learning and teaching of healthcare professionals and to provide examples of their use in context	No intervention, review—critical summary of key instructional strategies, learning objectives and evaluation approaches	Table of easy-to-use categorization/summary of education pedagogical methods, including recommendations for their application in Healthcare Edu settings	n/a	Learning theories, content and student understanding should be integrated to improve student learning.
**Strang AF et al. (2016) USA, review** [11]	Faculty staff and residents (published, peer-reviewed data synthesis on faculty Teaching development programs 2001–2015)	Intervention found: 1 of 21 focused on faculty development, 20 programs focused on resident teaching programs	20 interventions used subjective evaluations (participant survey), only 1 included an objective evaluation by an expert panel using validated tool	Yes	Program efficacy must be associated with better teaching ability, with a positive impact upon students (motivation, engagement, grades).
**Koster A et al. (2017) The Netherlands, review** [13]	Structural framework description, detailed, theoretical and classifies formal training and development programs	Complex for the degree course—design principles and adopting an explicit educational model, based on evidence-based educational psychology used in curriculum development and optimization. Conscious decisions on all organizational levels to achieve consistency between learning tasks, feedback to students, teacher roles and organization of the curriculum	n/a	Yes—other faculty experts serve as consultants to teachers	Successful implementation of CBPE requires a system of effective quality management and continuous professional development as a teacher.
**Stein SM et al. (2012) USA, study** [10]	Create template for presentations/lectures, course participants: College of Nursing educators, College of Pharmacy educators, panel: College of Education experts	One day teaching methods course including pedagogy, learning theory, teaching practice	Pre- and post-course video-recorded teaching presentations objectively assessed by an expert panel, using a validated evaluation tool. Participant pre- post-survey; objective results: significant improvement in 7 of 10 domains of teaching effectiveness	Yes	A short teaching-methods course can improve teaching effectiveness through enhanced communication and teaching. Training seminars can be integral to comprehensive quality improvements.
**Baia and Strang) (2016) USA, study** [18]	192 faculty and staff members employed at pharmacy schools with teaching roles before and after completing the HELP program—(2010–2014) survey—(online pedagogical professional development program)	Online professional development program titled Helping Educators Learn Pedagogy (HELP)	Qualitative and quantitative data analyzed for themes of motivation (data from written narratives, post-module quizzes and survey) converted into units and coded	n/a	Faculty educators must first value pedagogical knowledge for their continued growth as teachers, and the faculty development programs must appeal to the value of learning, wish to improve student learning and to educators’ beliefs regarding their roles and responsibilities.

## 4. Results

**Figure 1:**
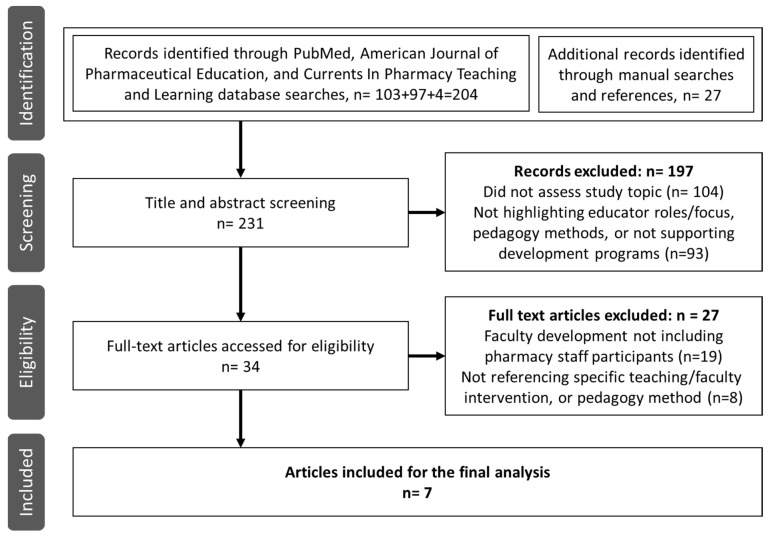
Flow chart of literature screening and the selection process.

In the identification process, the first literature search (n = 204) and additional searches (n = 27) resulted in a total of 231 records in support of the screening phase (see Figure 1). The titles and abstracts were filtered for academic pharmacy faculty development programs to identify implemented projects for the educators of undergraduate student pharmacists. During the title and abstract screening, 104 records were excluded for irrelevant topics and 93 for an irrelevant focus. A total of 34 manuscripts met the eligibility criteria for full-text analysis following a thorough content analysis. The two-stage eligibility process excluded many publications for not matching the first criteria of faculty development, including pharmacy staff participants (n = 19), or the second criteria of the integration or use of a pedagogical tool or approach (n = 8). Excluded papers consisted of white papers (n = 3), reviews with no referenced pedagogy interventions (n = 4) and research studies (n = 20). Several excluded papers focused exclusively on residency or seasonal staff (n = 8) or student performance following changes in course methodology without referencing educator development (n = 4). Of the interventions that did specify faculty development (n = 10), more than half (n = 6) were excluded for the absence of a pedagogical tool or approach.

The final seven articles comprised original research studies (n = 4) and reviews (n = 3) (See Table 1). All results (n = 7) either included or referenced faculty pedagogy knowledge development. Two reviews focused on an adult education intervention design and synthesized pedagogical theories and practice [16] or competency-based curricular development [13]. The other results mapped data for intervention effectiveness [10,11,16], including motivation factors [1,11]. An objective assessment of faculty development was detected in a single study [10]. We found data on interdisciplinary cooperation between pharmacy and educational faculties in three (42.8%) publications in the form of select membership specialization in didactic areas [13] and cooperation for the development design and pedagogy component [16] or throughout the intervention process [10]. The following nine synthesizing statements allow for a more focused presentation of the results.

**Statement 1: A change in faculty behavior focusing on peer support and interpersonal cooperation is required for curricular transformation**

An intervention by Edwards et al. in 2014 challenged faculty educators to incorporate at least one “new-to-you” teaching method in a class, course or experiential activity. Individual reports on the teaching experience were shared through brief presentations during monthly faculty meetings. The colleagues disclosed the new experience regardless of their proficiency level or methodology and felt comfortable to include what did not work as expected among “near peers”, which the authors considered a major achievement of the study [1]. They believe this educator openness also stems from the departmental efforts to elicit a mindset change among educators and shift focus to continuous quality improvements. The efforts were introduced a decade earlier, including a mandatory, formative peer-faculty evaluation of teaching and the demonstration of various teaching strategies by faculty opinion-leaders. The authors detected evidence for the broad recognition of measuring learning outcomes because all teaching challenge participants purposefully included some form of a post-intervention assessment, which, as the authors highlighted, suggested the educators developed a culture of openness and continuous quality improvements. Evidence of individual efforts for teaching proficiency significantly contributed to the successful intervention outcomes as, the authors also emphasized, many educators had adopted several new techniques even prior to the call for the challenge [1]. The study authors also recognized the potential benefit of peer support given the high percentage (63%) of educators who found inspiration from the peer presentations. The authors concluded that the intervention successfully initiated faculty development, enhanced curricular improvement and introduced elements of student-centered teaching. Stein and colleagues reported that successful mentoring creates a relatively cheap and easy networking system within a faculty when it is kept simple and informal [10].

Mukhalalati and Taylor, in the Journal of Medical Education and Curricular Development (2019) [17], recommended that health professional faculty behavior may be enhanced by a flexible network of peer support, which can optimize staff competency and enhance educator proficiency. Faculty interaction should be encouraged to balance the varying levels of educator teaching expertise and reduce stress levels of young or new educators in the health educator position. The authors [17] report that pharmacy educators receive no formal training prior and acquire teaching skills through experience, which necessitates more peer support, the exchange of professional expertise and resources, as well as new faculty development opportunities in support of their integration as academic educators. The study shared the views of McAllister et al. [19], who underlined that interprofessional cooperation will promote educator satisfaction and positively impact student learning experiences. Since curricular design is essentially a creative process, Koster and colleagues inferred that interpersonal cooperation between dedicated stakeholders, including teachers, students, educational specialists and administrative staff, is optimal to ensure effective curricular transformation [13].

**Statement 2: Competency focus is a key element of curricular reform**

Competency-based education and training requires consistency at all levels in the educational process. Koster et al. recommend the early introduction of professional competencies in building blocks and their gradual integration and development using problem-based methodology throughout the pharmacy curriculum. These blocks integrate knowledge, skills, values and attitudes expressed as professional behavior, later transferred into teaching and learning activities, which all educators must learn to recognize. The same authors also propose that a new approach should be used for the optimalization of the modern undergraduate curriculum in preparing young pharmacists for the expected professional skills in working life. The new approach recognizes the complexity of competency-based pharmacy education to promote a seamless transition into advanced pharmacy practice or postgraduate training programs. Also, educators must possess adequate pedagogy knowledge and gain professional insight into how skills development is organized, monitored or assessed. The study claimed that any isolated or individual educator development efforts tend to be ineffective; therefore, faculty members should receive comprehensive development preferably with educational expert involvement [13].

Stein et al. suggest that educator proficiency positively impacts student academic performance, which infers that educator competency development should be adequately measured. On the other hand, based on the reviewed literature, we deduced that participant self-evaluation is often the only assessment for measuring faculty intervention outcomes [1,11,16,18]. Studies that measure experiential component effectiveness in faculty development programs are scarce [11]. Johnson et al., alone, detected scarce student feedback on participant teaching performance, thereby allowing for the post-interventional self-perceived quiz on participant content knowledge development to be more objectivity supplemented [16]. Only a 1-day course in 2012 by Stein et al. integrated multiple approaches to evaluate experiential performance. Participant presentations were evaluated by an expert panel using a formal evaluation tool. The objective assessment included an evaluation of student gains from each educator’s presentation.

**Statement 3: Educational expert cooperation can ensure relevant pedagogy and reliable implementation outcomes**

The scientific value of interdisciplinary education opens a new window for the integration of skills with new curricular content. Koster and colleagues underlined that educator skills development and the exchange of specialist knowledge between academic faculties or institutions can promote the suitable adaptation of student learning activities [13]. To illustrate its implementation, the authors presented an example regarding a university in Utrecht, The Netherlands. Select teachers were encouraged to specialize in five didactic areas, including oral and written communication, compounding, research methodology or pharmaceutical calculations, and established interdisciplinary cooperation with other local faculty or university educators. This networking distributed scientific knowledge to ensure the integration and translation of new student skills and content knowledge into adequate teaching and learning activities throughout the curricular update. The teachers were assigned more responsibility and served as consultants to the course coordinator colleagues.

In a different study at Shenandoah University’s School of Pharmacy by Johnson et al., didactic experience became a core element of a teaching certificate program after interdisciplinary cooperation was established between the school of pharmacy and the school of education and human development. The program offered scientific knowledge development through educational expert mentorship during the didactic seminars to support the preparation of individual teaching philosophy statements. The educational experts ensured content reliability for the teaching philosophies, which were included in the didactic documentation and were formally evaluated in the participant’s teaching portfolio [16]. A detected study that showed the most comprehensive integration of educational expert cooperation was authored by Stein and colleagues [10]. The intervention integrated aspects of pedagogy and learning theory and included an experiential component. The course participants (n = 12) were recruited faculty members from nursing (n = 3) and pharmacy (n = 9). The 6 h didactic course provided education on effective teaching strategies, supported individual template development for effective lectures, and introduced the educators to active-learning techniques in pairs and small groups. Each participant prepared a presentation during the course. The interdisciplinary panel of educators with teaching excellence represented pharmacy, nursing and the education disciplines, received on-site training and validated the evaluation tool. As a result, 10 of the 16 assessment criteria were selected matching the project design.

**Statement 4: The flow of information between stakeholders can ensure curricular effectiveness and optimize student benefits**

The different aspects of information exchange were underlined in the reviewed studies. Koster et al. deduced that the role of continuous communication should be highlighted between all stakeholders, from intervention design throughout the full curricular change [13]. As the authors illustrated, this consistency can prevent excessive student burdens and ensure that students will remain the main beneficiaries of the transition. The review authors articulated that sharing a vision of future goals with both teachers and students will significantly impact the resulting effectiveness of a new curriculum. Additionally, two factors enhance seamless transition: a demand for change both outside (professional organizations) and inside (teachers, students and alumni) of academia, and faculty leadership is encouraged to launch a fundamental change. The review authors emphasized that faculty members need to be aware of the potentially new focus and responsibilities, approve them and actively participate in the transition process, including faculty development programs [13].

The significance of information exchange between faculty leaders and members for a more student-centered education was also emphasized by Edwards et al. [1]. The authors suggested that the open faculty meetings produced broader involvement, which the authors believe illustrates faculty efforts for clear communication and the recognition of collegial support. Stein and colleagues postulated that ineffective teaching may become less common “if faculty members were more frequently encouraged to openly discuss teaching-method successes and failures” [10].

**Statement 5: Faculty engagement factors impact program efficacy and curricular development**

Two papers [1,18] aimed to map faculty motivational factors in seeking development opportunities to attain pedagogical knowledge and educator proficiency. The voluntary 50 h online summer teaching development program (HELP-Helping Educators Learn Pedagogy) at Albany College described by Baia and Strang in 2016 [18] provided educational strategies through video shares and research websites and offered a platform for peer discussions to promote educator engagement. The study assessed motivational factors using a post-module quiz and open-ended narrative surveys. Pre-intervention narratives on the motivations for joining the program showed no external factors (e.g., funding, promotion), only the goal towards becoming better teachers to promote student learning. The modules developed new knowledge in teaching theory, lesson planning, assessment, teaching methodology and teaching technology. Results reported that the strongest motivations were an intrinsic passion to learn and help students learn. Similar results by Edwards et al., at Northeastern University, showed [1] that the top three factors for joining the intervention included the motivation “to improve student engagement”, “to improve student learning” and “to improve teaching”. The post-interventional survey showed plans to continue joining the HELP program and the significance of continuous pedagogical knowledge development [18]. The study proposes that successful faculty development programs need to address individual core beliefs, participant values of learning and the roles and responsibilities as an educator. The authors postulated that faculty members expect both the program content and learning environment to be appealing, useful and reliable. The online platform and flexible summer schedule likely increased the participation rate [18].

Edwards et al. showed that motivation factors and a tailored course design may be important points for educational research to address faculty development involvement [1]. The authors conducted a target search in adult education literature and found that adults strongly prefer self-directed learning. Therefore, a flexible intervention design was used in which adult educators selected the educational settings for introducing the new teaching method. Pre- and post-intervention surveys were used to assess changes in teaching competency, yet student feedback was not collected. Here, 96% of the educator participants found the intervention to be useful and indicated their participation the following semester. Stein et al. [10] also believe that educator motivation is important and may be improved through informal discussions during faculty interventions. Their intervention design purposefully included an informal lunch during the intervention to stimulate participant networking and encourage educator discussions about their teaching experiences.

**Statement 6: Apply relevant evidence-based data on adult teaching pedagogy and adapt to individualized educational settings**

An invaluable article drafted by Mukhalalati et al. [17] provided an excellent tool for exhausted health professionals to improve daily teaching or take steps towards adopting a more educational approach. The review details an objective critical summary of adult learning theories with recommendations on their optimal application in health educational settings (medicine, nursing and pharmacy). The listed results offer educator guidance for matching educational settings with the myriad of pedagogical strategies. Despite numerous articles regarding education development, this recent publication clearly indicates that health educators still seek relevant, adaptable and evidence-based data on adult teaching pedagogy. This knowledge, as the authors concluded, should be applied and integrated and become common practice within the health educator community. Edwards et al. also underlined educator responsibility for the attainment and adaptation of suitable pedagogy knowledge [1], with the intervention that aimed to encourage educator initiatives by promoting access to all institutional educational resources [1].

Johnson and colleagues reported how pedagogical knowledge and an experiential component may be linked to professional mentorship within a teaching certificate program. The newly introduced twelve didactic seminars integrated design and cooperation, with educational experts providing foundational pedagogical knowledge. Each seminar was recorded to ensure subsequent access and dissemination of the theoretical knowledge, which indicates its significance in the program. The experiential component also received more weight and included two lectures prepared through continuous content expert mentorship. Participant performance was recorded to allow self-reflection and a formal evaluation [16].

**Statement 7: Recognize and develop the informal (hidden) curriculum**

Based on the constructivist learning theories, as Koster and colleagues pointed out [13], the quality of student learning is not dependent only on the disciplinary content, as in stereotypical “traditional” teacher-focused education. The authors elaborated that competency-based education outcomes are strongly impacted by the teacher’s character, delivery of teaching, motivation and behavior, both inside and outside the classroom, through the informal curriculum. Insightful adaptation of the informal curriculum was illustrated with a lead-by-example design in which active-learning methodology was used within faculty pedagogical development, as described by Stein et al. [10]. The course aimed to improve educator teaching effectiveness by implementing active-learning strategies to achieve an impact on student learning outcomes. Thus, course objectives and content were introduced with interactive discussions, which allowed educator participants to receive first-hand experience of a methodology and its resulting audience impact. The usefulness of the lead-by-example approach when adapting active-learning strategies for a faculty development program was underpinned by the promising long-term results when several educator participants, one year after the intervention, perceived improvements in teaching proficiency and reported significantly improved end-of-course student feedback rates.

Edwards et al. also recognized the link between educator methodology and the hidden curriculum suggesting that educator relatedness can improve through pedagogical knowledge development, which is strongly linked to educator recognition of what students may go through during the learning process. The program objectives included raising awareness of student classroom experiences to improve educator relatedness and teaching proficiency [1].

**Statement 8: Reflection on learned or experienced pedagogical knowledge is a key element when implementing theory into practice**

The literature shows that teaching portfolios are commonly used to raise awareness of pedagogical knowledge focusing upon future educators. It is not surprising that Strang and colleagues detected that 40% of the reviewed interventions required participants to formulate their own teaching philosophy and 50% to develop their teaching portfolios [11], since educational philosophy and learning theory underly all educational settings and practices [17]. A teaching certificate program updated by interdisciplinary cooperation encouraged a more professional pedagogical approach, as described by Johnson and colleagues [16]. The pedagogy seminars were facilitated by the educational experts who raised educator awareness through mentorship during the one-year course. Koster and colleagues also noted the significance of prior teaching experiences in collaborative development efforts for improving educator teaching competency. The authors stated that individual reflection and the acknowledgement of educational research results promote the transition towards a more competency-based education [13]. Edwards et al. identified the intervention design that was based on the educators’ reflective ability based on educational experiences [1]. The faculty had prior access to academic educational workshops and listened to the novel methodology for peer classroom experiences. The intervention participants were to integrate a prior workshop and new peer presentation experiences with the individually selected innovation strategies, thereby linking theory with practice. Mukhalalati and Taylor (2019) clearly conclude that the delivery of teaching is inevitably related to the underlying theoretical considerations [17].

**Statement 9: Adoption of the educator role of a facilitator, motivator and formative assessor encourages student progress**

Based on the current research results, Koster et al. clearly articulate that the professional educator’s key role today is to act as a facilitator of student learning and optimize the learning environment. Competency-based education is undermined by constructivist psychology theories, which focus on student learning, the learning environment and the activities through which new knowledge is acquired [13]. On the other hand, an educator is responsible for matching the educational settings (‘where?’) with a suitable design (‘how?’). The authors also suggested that educational literature provided ample evidence, in which not only the student’s cognitive, personality and motivation factors, but the teacher’s behavior, will also directly (both positively and negatively) impact the quality of student learning [13].

Academic educators also play an important role in the design and implementation of student assessments [13]. Formative assessments (e.g., in-class discussions or homework assignments evaluating the method of learning) and summative assessments (e.g., oral or written tests evaluating learning outcomes) should be distinguished and applied based on their function. As the authors pointed out, more complex summative assessments (usually during the senior years) should be carefully designed and limited in number to avoid overburdening the students. On the other hand, the consistent use of formative assessments, which monitor the learning process instead of testing knowledge, is designed to provide progress feedback to both students and educators. The importance of formative tests is neglected in the literature; they deserve more focus and require more creativity for task development (e.g., games, portfolios) [13].

Mukhalalati and Taylor summarized the complex roles of academic educators and proposed that new teaching strategies, objectives or assessments should be preceded by attaining relevant theoretical knowledge. Theoretical considerations play a significant role in any professional healthcare education, as underlined by Benner et al. in Mukhalalati and Taylor (2019), who suggested that “theoretical knowledge is formed by practice and consequently influences practice” [17], which further underlines academic educator responsibility as health professionals and supports the implementation of key learning theories in health professional development programs.

## 5. Discussion and Illustration of a Professional Pedagogical Knowledge Development Program

In our scoping review, we aimed to detect faculty efforts for comprehensive and sustainable development, which ensure continuous mapping between curricular outcomes and entry-level practitioner competencies. We hypothesized that educator teaching proficiency forms a key element and is developed through interdisciplinary cooperation in many reported interventions. Prior studies did report moot suggestions and unmeasured evidence of interprofessional collaboration within faculties [20,21], yet our hypothesis was not confirmed since only a few detected interventions that involved educational scientist support for the pedagogical development at any stage of the process [10,13,16]. Institutional awareness of interdisciplinary cooperation benefits should be raised and more aptly documented.

All seven publications used or integrated a certain degree of pedagogical knowledge development, including learning theory and teaching strategies in undergraduate pharmacy education, and were complemented by experiential components, which encourage a deeper understanding, enhance reflective ability and ensure improvements in teaching competency [22,23].

Based on the reviewed literature, competency focus remains a key element in the curricular reforms of pharmacy education. The located studies reported on the implementation aspects of competency-based education [13], student-learning outcomes or faculty experience with the use of team-based learning [15,24,25,26] or active-learning methodology [27,28] or introduced complete competency-based frameworks [13].

The reviews of 2001–2020 faculty programs identified the need for more objective measures of intervention outcomes impacting staff, students or the university [9,11,29]. Our single included study [11] integrated a validated formal evaluation tool and objective panel assessment through educational expert cooperation suggesting the scarcity of such data.

Only one publication [10] showed extensive educational scientist cooperation, which indicates that the benefits of interdisciplinary cooperation are still underrated or unrecognized. We propose that the integration of pedagogical knowledge development and relevant expertise play a key role in ensuring optimal intervention results. Based on extensive literature [10,13,15,17,18,23,27,30,31], we propose that teaching delivery methods should be selected in optimal support for the given course objectives, since educator strategies will impact student experiences, student learning and the rate of involvement, thereby influencing course effectiveness. The reviewed studies propose that active-learning, or more “student-friendly” teaching strategies, enhance student learning experiences and effectiveness. Further, educator teaching strategies reflect the informal curriculum, which forms an integral yet often unrecognized and underrated element of any education. We wish to present a model to illustrate an intervention design of a faculty development implemented in close cooperation with educational faculty experts. The model also serves as a real-world example of our Results Statements by presenting a coherent, comprehensive and interdisciplinary model of faculty development in Hungary. In Table 2, we highlighted the corresponding points between the Results Statements and our intervention. Our curricular and pedagogical development used a framework incorporating elements of the various procedural learning models of Kotter [32,33], SMART [34], 5E model [35,36] and Gillies [6]. These models are aligned with two implementation science models: the Normalization Process Theory [37], which focuses on social processes integrating new practices into existing routines, and the Diffusion of Innovations theory [38,39], which emphasizes the features and adopters of an innovation and the social networks impacting adoption. Although these models were developed for healthcare procedures, their cognitive and social approach are crucial for educational intervention implementation. In line with our findings, which underscore the significance of faculty courage for initiating change, it was a change in faculty leadership attitudes that launched the curricular development process in 2019 (Statement 1) in collaboration with the educational faculty. Three higher education pedagogy experts from the Institute of Educational Sciences of the Faculty of Humanities and Social Sciences of the same university were invited to design, implement and coordinate the developmental process. Supervised by a newly established faculty committee, a curriculum audit worksheet was prepared for the design process, which allowed for a complex approach and analysis of the curricular reform. Leadership engagement and supervision of the process illustrate their dedication to the development. The worksheet collected course data and placed a special emphasis on input and output competencies and their integration into professional knowledge, skills, values and attitudes, as described in Statement 2. The education experts possessed extensive professional experience in the tailored design and implementation of pedagogical development programs for academic institutions, both nationally and internationally, which ensured relevant pedagogy knowledge and reliable program outcomes (Statement 3).

Each institute was represented in the Faculty Development Board established to ensure transparency and the continuous flow of information between institutional stakeholders, including leadership and full faculty staff (Statement 4). The board identified the potential challenges (both general and specific), which launched an education expert faculty support service and promoted engagement via voluntary micro-groups (3–4 members) in each participant institute. The tentative goal to reach a minimum of two institutes was surpassed when as many as five of the nine institutes accepted the offered pedagogical service, which covered modifying a selected course element (for example, to complete each lecture with a metacognitive task) or even the coordination and complex development of lectures and seminars (Statement 5). The provided service tools matched the pedagogical approaches from the relevant literature, including digital technology, which allowed personalized mapping and a self-regulative learning process, which builds upon students’ prior knowledge. The tools also included course designs of cooperative learning structures with tasks that promote students’ cognitive development and learning or mobilize broad cognitive domains for processing lecture content (Statement 6).

The educational mentors assisted educators in selecting the most suitable pedagogical approach, methodological concept or even complete methodology for their individual challenges during the interpersonal or micro-group, face-to-face or online consultations, also offering evidence-based development opportunities and tailored guidance throughout the intervention (Statement 3).

Each development stage (preparation, trial and evaluation) spanned the full fourteen weeks of the semester. The preparation phase explored the hidden curriculum through the pedagogical views reflected in participants’ teaching challenges or difficulties (Statement 7). During the trial stage, assisted by introspective mentorship, educators were confronted with how the purposeful implementation of pedagogical ideas they wanted to consciously follow replaced their former ideas regarding teaching. Educators realized that they may also strengthen input improvement (e.g., expected prior student knowledge) using the online diagnostic and learning support tools for self-regulative development. Educators also realized that the underlying factors of student learning behavior include the behavior, selected tasks and pedagogy of a course teacher. The educators recognized their impact on student involvement through structural changes, higher and deeper knowledge transfer or the prior time-framed practice activities (Statement 8).

The evaluation stage disclosed a significant improvement when educators abandoned “old-school” scientific knowledge delivery and gradually became facilitators of scientific thinking and new knowledge. During the pilot phase, despite prior student passivity during lectures, educator initiation of self-regulative student learning processes brought significant improvements in attendance rates, levels of students’ inter-class preparation, in-class activity, involvement and general student behavior. On the other hand, the new course requirements (with a restructured scoring system) were not proportionately adjusted to the term assessment scores, which was shown by the surprisingly low performance average on final tests. Most students were satisfied with their gains through active course participation and preparation for interclass activities and thus neglected preparation for the final tests of the given course, as comparative performance results revealed. The facilitator’s direct impact on student behavior was clearly detected. The results were not all positive, yet educators still benefited from the experience since it successfully raised awareness for optimal transitioning design (Statements 3, 6, 7, 8, 9).

Another group of educators found only positive results. They used cooperative learning structures with significantly high rates of student involvement and proportionately higher performance rates in each study group while using the same evaluation tools as in prior years. During the evaluation phase, both negative and positive results were analyzed and effectively highlighted the need for the conscious development of educators’ pedagogical competence in promoting student engagement, thinking skills or academic development and, more importantly, their ability to facilitate the teaching and learning process (Statement 9).

Based on the first three development stages, the three pillars identified in the literature provide a suitable framework and ensure personalized educator support for the implementation of complex faculty development. The three pillars comprise (1) the voluntary micro-group cooperation of educators, (2) mentorship involvement of educational professionals as consultants and (3) full faculty management support. The three developmental stages of preparation, trial and evaluation are followed by the adaptation phase, in which the focus is shifted from educators to the measurable results of student performance. Our preliminary data, as illustrated above, focus on student and educator involvement. Objective intervention outcomes measuring student performance will be available and reported following the next development stages.

Our study exhibits both strengths and limitations. Potential limitations may stem from our exclusion of non-English language sources, as our search primarily targeted a comprehensive biomedical literature database and not educational databases (e.g., Education Resources Information Center, ERIC); the preference for manual search methods; and the strong focus on a specific target audience (graduate pharmacy students), which may have excluded study focus overlapping with other health sciences. Furthermore, the relatively low number of located publications suggest that our study’s focus remains within an under-researched area. Limitations to the relevance of our results may be due to scarce objective outcomes, since 90% of the located studies relied on sources of self-reported data. However, a notable strength of our study lies in the careful and extensive manual screening process, which effectively resolved any ambiguities and facilitated the precise identification of a unique and narrow intersection between pharmacy and education. Additionally, our research team brought together knowledge and expertise from various disciplines, enabling us to make a valuable scientific contribution to an international audience. We introduced, in the Discussion, the framework points of our ongoing intervention, which illustrated the real-world applicability of the Results Statements to provide further evidence for the implementation and potential benefits of an educator pedagogy knowledge development integrated in a comprehensive curricular development plan.

## 6. Conclusions

We were interested in locating recent peer-reviewed research publications that describe or review faculty development interventions using a pedagogical tool or approach from which undergraduate pharmacy students may benefit. Unfortunately, we found few papers focusing on this specific target population and even fewer with objective assessments of educator proficiency development, despite the articulated need in scientific discourse [11]. We also presented initial findings from an evidence-based, holistic faculty development framework, the efficacy of which will undergo a more rigorous evaluation in upcoming intervention phases. Should subsequent objective assessments validate the effectiveness of this curricular intervention, further studies will investigate the adaptability of the framework across diverse educational settings, potentially paving the way for a quantifiable and flexible model that can be applied in various higher education environments.

Interprofessional communication barriers seem to be a prevailing factor in the course of academic progress [20]. Yet, we propose, collaboration and mutual respect between experts of different sciences seem to be beneficial for sustainable quality improvements [20,21,40]. Important implications arise from the complexity of the curriculum, which integrates the full (formal, informal and hidden) curriculum. Educator teaching proficiency is embedded in the informal curriculum and strongly linked to student learning and professional skills development; therefore, educator proficiency and its continuous development should constitute an integral element of curricular transformation in pharmacy education and demand clear institutional support. Comprehensive curricular transitions in pharmacy education, integrated with faculty development, are seldom reported in the literature. However, multiple research findings suggest that a curriculum is not just a structural framework but rather functions as an organic entity. In this context, all stakeholders, including faculty leadership, administration, educator staff and students, should actively participate in the curricular update process. We advocate for complex development processes that provide a more holistic approach, as they are more effective in achieving long-term goals and ensuring sustainable curriculum development.

## Figures and Tables

**Table 2:** Application of review Results Statements in practice within the faculty interventions at the University of Pécs, Faculty of Pharmacy, between 2019 and 2023.

Literature Review Findings Summarized as Numbered Results Statements	Faculty Interventions and Implementation of Results Statements
1. A change in faculty behavior focusing on peer support and interpersonal cooperation is required for curricular transformation (Statement 1)	Faculty leadership initiative: competency-based curriculum audit, initiating a cooperative organizational structure for faculty development
2. Competency focus is a key element of curricular reform (Statement 2)	Emphasis on input and output competency elements: detailed elements of knowledge, skills and attitudes elaborated throughout the curricular audit process
3. Educational expert cooperation can ensure relevant pedagogy and reliable implementation outcomes (Statement 3)	Involvement of education experts with higher education pedagogical and institutional development experience
4. The flow of information between stakeholders can ensure curricular effectiveness and optimize student benefits (Statement 4)	Setting up Faculty Development Board and Developmental Micro-groups of educator participants emphasizing transparency
5. Faculty engagement factors impact program efficacy and curricular development (Statement 5)	Personalized support: identification of challenges; wide-spectrum educational counselling services for faculty staff to provide personalized engagement and support
6. Apply relevant evidence-based data on adult teaching pedagogy and adapt to individualized educational settings (Statement 6)	Demonstration/Application of broad repertoire of pedagogical service tools and approaches from evidence-based literature introduced by experienced professionals
7. Recognize and develop the informal (hidden) curriculum (Statement 7)	Preparation phase: exploration of the hidden curriculum and educator views on teaching and learning through the articulated educational challenges
8. Reflection on learned or experienced pedagogical knowledge is a key element when implementing theory into practice (Statement 8)	Trial phase: effectiveness based on instructors’ self-reflection, student behavior, feedback and performance supported by individual and micro-group periodic pedagogical expert consultations
9. Adoption of the educator role of a facilitator, motivator and formative assessor encourages student progress (Statement 9)	Evaluation phase: raising educator awareness of the relevance of teaching proficiency development based on Developmental Micro-group cooperation and transparency of the complete developmental process

## Data Availability

Data supporting the findings of this study are available from the corresponding author AF, Andras Fittler, on request.

## Supplementary Materials

The following supporting information can be downloaded at: https://www.mdpi.com/article/10.3390/pharmacy11060172/s1, Table S1: Manuscripts eligible for content analysis [30,41,42,43,44,45,46,47,48,49,50,51,52].
