# Supplementary material for: A Scoping Review of Educator Proficiency Interventions in Pharmacy Education Illustrated by an Interdisciplinary Model Integrating Pedagogical Theories into Practice
Source: Pharmacy (Basel). 2023 Oct 30;11(6):172. doi: 10.3390/pharmacy11060172 (PMC10661261; doi:10.3390/pharmacy11060172)
Supplement: Supplementary file 1 [file pharmacy-11-00172-s001.zip › pharmacy-2658370-supplementary.pdf]

No.	Authors, (date), country and publication category	Participants (population)	Pedagogy method used or referenced (intervention)	Evaluation of the results (outcome)	Are pharmacy students beneficiaries of the intervention, or will likely benefit?	Evaluation of effectiveness by undergraduate students, or students' performance	Key findings	Interdisciplinary cooperation	DOI
1	**Johnson MS et al. (2013) USA, study [17]**	Participants were pharmacy residents, new faculty, residency preceptors; new program component introduced formal pedagogy seminars (priorly: only monthly discussions) with educational expert cooperation providing foundational pedagogy knowledge	Pedagogy seminars (12 lectures), didactic components required 2 participant lectures, small group facilitation, experiential teaching, development of teaching philosophy statement and teaching portfolio	Participant subjective evaluations included pre-and post-intervention feedback on program benefits, formal training and experiential performance; formal objective evaluation of participant experiential performance was given by mentors based on objective pedagogy assessment factors	Yes - (undegraduate students are impacted)	Not comprehensively - but student feedback was provided regarding resident's performance in the experiential component	Validity and relevance of pedagogical knowledge was ensured by educational experts in the design, scheduling and facilitation of didactic seminars. A joint school of pharmacy and school of education program has increased resident-perceived teaching abilities and confidence.	Yes (in pedagogy seminars)	https://doi.org/10.1016/j.cptl.2013.02.004
2	**Berthod F et al. (2020) Switzerland, study [41]**	Participants (N=72): 29% senior pharmacists, 14% junior pharmacists, 57% pharmacy technicians; the intervention aimed to evaluate innovative teaching tool impact on pharmacists' GMP knowledge and confidence, and survey tool satisfaction	The pedagogical objective was to change participant traditional approach to teaching. Pairs of participants were locked in a simulated clean room to solve 23 'Good Manufacturing Practices mysteries' on the topic of producing a chemotherapy in an escape room simulation.	participants completed a 23-item online questionnaire which rated improvement in GMP knowledge, confidence and perceived appreciation of new tool prior and one month post intervention	No - (undegraduate students were not impacted)	No	All participants would recommend this teaching method to their staff colleagues. Results showed increase in correct answers and certainty. Correct answers rose from 57% to 80% in the third questionnaire. Certainty scores rose from 50% before the experiment to 70% one month afterwards.	n/a	DOI: 10.1177/1078155219875504
3	**Edwards RA et al. (2014) USA, study [1]**	Motivate pharm. educators in improving teaching methodology, practical development through sharing peer practical teaching experience	A 'new-to-you' teaching method was shared, no staff pedagogy education included in the faculty intervention.	Self-evaluative qualitative feedback survey	Yes	No	A teaching challenge motivated most of the faculty members to try something new. Links between evidence-based principles and day-to-day activities were strengthened by the peer-teaching method.	n/a	10.5688/ajpe785103
4	**Hammer D et al. (2010) USA, study [40]**	The study reviewed pharmacy school good practices and identified evidence-based criteria for the definition of teaching excellence for its better acknowledgement.	Based on research data, assessment criteria for good teacher skills include 1. Positive student-faculty contact 2. Effective active-learning methodology 3. Achievable, yet high expectations 4. Respects diverse talents and ways of learning 5. Effective communication skills 6. Commitment to teaching well.	A narrative presentation of the review results. Teaching excellence evaluation may involve student/ alumni, peer, technician or/and educator self-perceived evaluation.	n/a	n/a	All educator faculty members should engage in professional development to improve their teaching, with an emphasis on a scholarly, collaborative approach.	n/a	10.5688/aj7409164
5	**Kennedy DR et al. (2020) USA, commentary [42]**	It provides recommendations to pharmacist professionals also in academic educator position regarding the design, conduct and publication process of education-related scientific research.	The commentary provides general advice for those who wish to explore Scholarship of Teaching and Learning but lack formal education and training in the area.	It highlights four opportunity areas for researchers: small activities and projects, course redesign, and longitudinal assessment and evaluation.	In the authors' experience, collaboration, willingness to ask questions, fail and learn from mistakes, intrinsic drive, grit and determination, and ability to adapt one's skillset and talents can help position the scholar for success in SoTL.	n/a	Suggests a shift to multi-institution samples, from pharmacy-centric to interprofessional perspective in methods, sampling, and publication.	n/a	10.5688/ajpe7702

No.	Authors, (date), country and publication category	Participants (population)	Pedagogy method used or referenced (intervention)	Evaluation of the results (outcome)	Are pharmacy students beneficiaries of the intervention, or will likely benefit?	Evaluation of effectiveness by undergraduate students, or students' performance	Key findings	Interdisciplinary cooperation	DOI
6	**Knott G et al. (2015) Australia, review [29]**	It focuses on sessional staff : tutors, demonstrators, graduate teaching assistants (GTAs), casual academic staff, and teacher practitioners and maps the benefits, structure, and content of training programs.	Educational training program components included understanding student learning, communication and presentation skills, group facilitation techniques, managing and motivating students, student feedback, assessment and grading.	most detected interventions only assess Kirkpatrick's Level 1-2 (reaction, learning) effect on participants, only few studies investigate L3 (behavior), L4 (results) program impact regarding work performance, student engagement, or student outcomes	The three program components comprised of orientation, educational issues, tutor facilities and ongoing support.	n/a	The review detected a widespread lack of follow-up professional development and support.	n/a	10.5688/ajpe79572
7	**Lonie JM and Desai KR (2015) USA, review [43]**	It describes the review and application of transformative learning theory (TL) in a classroom or clinical setting and recommends its practical use in professional skill and self-development of both students and educators.	Transformative Learning Theory from constructivist learning theories focuses on introspective techniques to support self-awareness and understanding of one's own cognitive processes which may be used to develop pharmacist patient-centered skills and educator self-development.	A narrative presentation of the review results.	Yes, because TL can improve decision-making and students can become more autonomous as individuals and health professionals.	n/a	The use of TL can help challenge the validity of one's underlying assumptions by promoting a change in points of view and habits of the mind. Self-reflection may support the development of an autonomous individual.	n/a	https://www.infona.pl/resource/bwmeta1.element.elsevier-0d2ad75f-82ac-3250-a560-2c6ed10e5d4d
8	**Lucas KH et al. (2013) USA, study [28]**	The intervention measured the effectiveness of a pharmacy school's new pedagogy. It implemented an active-learning approach in a pharmacotherapy course sequence in the second year (P2) and third (P3) year of a doctor of pharmacy (PharmD) program to determine whether the pedagogical changes correlated with retention of core content in the fourth year (P4). The study compared student retention rates of conceptual knowledge based on student scores from a comprehensive written exam.	Traditional slide-based teaching was used in yr 1, and discussion-based active-learning methodology was used through yr 2,3 in the sequence of pharmacotherapy course.	Subjective assessment included student feedback, pre- and post-intervention faculty survey evaluating educator attitutes using active-learning methods. The objective 4th yr 100-item student exam included 30 questions on each course testing knowledge, comprehension and application based on Bloom's Taxonomy levels.	Some faculty members also introduced aspects of active-learning pedagogy in other courses. Student exit interviews reported greater sense of responsibility, and improvement in self-learning skills.	A shift from lecture-based teaching to discussion-based active-learning pedagogy reported changes which correlated with the retention of core content in the fourth year (P4).	Both faculty and student barriers reported extra preparation or pre-class reading time (students expressed complaints as 'teaching ourselves'), but overall feedback showed positive results. Student perceptions included a resulting deeper understanding of the material.	n/a	10.5688/ajpe778171
9	**Mukhalalati BA et al. (2019) Qatar, review [16]**	Literature review using term 'andragogy'; to synthesize key learning theories applicable in the learning and teaching of healthcare professionals and to provide examples of their use in context	No intervention, review - critical summary of key instructional strategies, learning objectives, and evaluation approaches	Table of easy-to-use categorization/summary of education pedagogical methods, including recommendations for their application in Healthcare Edu settings	Yes, (if educators use it): Edu and learning theories: essential in evidence-based edu practice, for selection of best strategies, objectives, evaluation for matching setting.	n/a It is important that educators explicitly explain the benefits of implementing graduate pedagogies in HP education programs.	Learning theories, content and student understanding should be integrated to improve student learning.	n/a	DOI: 10.1177/2382120519840332

No.	Authors, (date), country and publication category	Participants (population)	Pedagogy method used or referenced (intervention)	Evaluation of the results (outcome)	Are pharmacy students beneficiaries of the intervention, or will likely benefit?	Evaluation of effectiveness by undergraduate students, or students' performance	Key findings	Interdisciplinary cooperation	DOI
10	**Schwartz A et al. (2018) USA, research brief [44]**	The study aimed to determine professionalism factors in a school of pharmacy (USF) and develop a plan to monitor professionalization of students, faculty staff and preceptors.	Key professionalism factors were identified by school research group for incorporation in pharmacy practice experiences, didactic courses, faculty and preceptive performance evaluations.	The Behavioral Professionalism Assessment Form-Experiential (BPAE) validated scoring system was used to assess faculty and preceptor perceptions. Factor analysis results led to faculty discussions resulting in an objective plan for professionalism evaluation.	Broadened evaluation focus involved admission, student affairs, student assessment and staff evaluation.	not measured - "Hopefully the proposed programmatic plan will further support student professionalization, foster the continued professionalization of faculty, preceptors, and staff."	Degree program inconsistencies tend to inhibit degree course development.	n/a	10.5688/ajpe6478
11	**Sicat BL et al. (2014) USA, study [20]**	The study described the collaboration of five Health Sciences schools of the same university, which formed an interprofessional, voluntary HEI faculty learning community (FLC) to coordinate faculty development projects. The study presented the FLC program design, progress and perceived outcomes.	FLC program participants included medicine (8), pharmacy (2), dental hygiene (1), occupational therapy (2), and nursing (1) disciplines, shared expertise and resources every 6 weeks to promote inter-faculty collaboration. One of the workshops invited an expert to speak about effective clinical teaching in support of overlapping projects.	Feedbacks were collected on participant satisfaction regarding faculty development projects. A specific FLC professionalism workshop had over 200 HEI attendees and results showed 88% satisfaction with program relevance.	The quality of teaching was improved through workshops and sharing an online tool for Bloom's taxonomy learning objectives.	not measured	A participating educator feedback stated: "FLC has helped each of us to grow in ways that would not have been possible without the benefits of working together as a group."	n/a	10.5688/ajpe785102
12	**Sturpe DA and Schaivone KA (2014) USA, study [45]**	The study described the implementation of the Objective Structured Teaching Exercise (OSTE) training program as part of comprehensive faculty development for pharmacy faculty members and preceptors.	The OSTE training program integrates standardized students to support the development of the teaching skills and interpersonal communicational skills of educators and preceptors specifically with case presentations. Session issues include group teaching, individual teaching or mentoring and professionalism or behavioral issues.	The development of case scenarios, recruitment and training of standardized students, the implementation of an OSTE session and the evaluation of OSTE methods were presented.	Educator teaching and precepting skills may be enhanced and assessed using the OSTE method. It stimulates continuous quality improvement and update on relevant research from participants.	not available	Standardized student encounters may be used for instruction and practice of teaching skills, performance evaluation, program assessment and research. It allows unlimited repetition of the experience.	n/a	10.5688/ajpe785104
13	**Zagar M et al. (2019) USA, study [46]**	The study described faculty staff opinions on using a year-long book club format in support of a comprehensive pharmacy faculty development.	The faculty completed reading an educational text which stimulated interactive discussions during the monthly meetings. Discussion topics included educator roles, teaching skills development and scholarships.	Pre- post- participant surveys were collected regarding attitudes and opinions of faculty development using a book club format.	n/a	n/a	Interactive learning through peer discussions, and increased camaraderie were found the strongest factors for faculty engagement.	n/a	doi.org/10.1016/j.cptl.2019.05.005

No.	Authors, (date), country and publication category	Participants (population)	Pedagogy method used or referenced (intervention)	Evaluation of the results (outcome)	Are pharmacy students beneficiaries of the intervention, or will likely benefit?	Evaluation of effectiveness by undergraduate students, or students' performance	Key findings	Interdisciplinary cooperation	DOI
14	**Meng X et al. (2019) China, study [27]**	The study presented the implementation of Self-study, Test, Question and Discussion (STQD) methodology session to improve student learning effectiveness and develop their learning skills. Active-learning and student-centered teaching methods were used in instrumental analysis and analytical chemistry courses. The longitudinal (4-yr) study measured impact on student learning outcomes based on test results and feedback from student participants (N:240).	Self-learning, peer-learning, co-learning, active learning, inductive teaching methodology and formative assessment were used in an integrated basic science curriculum to promote student centered teaching in pharmacy education.	Both qualitative and quantitative measures were used to evaluate inintervention outcomes, both of which showed positive results. Student perceived improved learning ability, communication ability and facilitated learning. Interventional student group final exam (4th yr) test scores and average course scores were higher than in traditional classes.	yes	Both objective measures (4th yr final exam test scores), and subjective student survey on learning process, skills development and learning enthusiasm were assessed.	The utilization of STQD in pharmacy education has the potential for individualized teaching.	n/a	10.5688/ajpe6505
15	**Strang AF and Baia P (2016) USA, review [11]**	Faculty staff and residents (published, peer-reviewed data synthesis on faculty Teaching development programs 2001-2015)	Intervention found: 1 of 21 focused on faculty development, 20 programs Focused on resident teaching programs	20 interventions used subjective evaluations (participant survey), only 1 included objective evaluation by expert panel using validated tool	yes - one study measured changes in teaching behaviour	yes - not measured	Program efficacy must be associated with better teaching ability, positive impact upon student Outcomes (motivation, engagement, grades)	Yes	10.5688/ajpe80459
16	**Sheehan AH et al. (2020) USA, study [47]**	The study describes the availability of teaching and learning curriculum (TLC) programs sponsored by US pharmacy schools and colleges and evaluate their adoption of best practice recommendations using a 28-item electronic survey.	Most program requirements included participation in TLC seminars, facilitation of small-group student discussions, providing didactic lectures, and development of multiple-choice assessment questions.	About 28% of TLC programs did not undergo programmatic assessment and continuous quality improvement on at least a semi-regular basis. In addition, only half of the programs formally evaluated the faculty and mentors involved in delivery of the TLC program.	yes	Fewer than half of the individual programs reported formal evaluation of resident teaching being done by students or peers.	Inconsistencies among individual programs were noted in required teaching experiences, participant evaluation, and ongoing programmatic assessment.	n/a	DOI: https://doi.org/10.5688/ajpe7803
17	**Koster A et al. (2017) The Netherlands, review [13]**	Structural framework description, detailed, theoretical, and classifies formal training and development programs	Complex for the degree course - Design principles and adopting an explicit educational model, based on evidence-based educational psychology used in curriculum development and optimization. Conscious decisions on all organizational levels to achieve consistency between learning tasks, feedback to students, teacher roles, and organization of the curriculum.	n/a	yes	n/a - Implementing CBPE requires ‘translation’ of formulated competencies into intended learning outcomes and assessment formats.	Successful implementation of CBPE requires a system of effective quality management and continuous professional development as a teacher.	Yes - other faculty experts serve as consultants to teachers	10.3390/pharmacy5010010

No.	Authors, (date), country and publication category	Participants (population)	Pedagogy method used or referenced (intervention)	Evaluation of the results (outcome)	Are pharmacy students beneficiaries of the intervention, or will likely benefit?	Evaluation of effectiveness by undergraduate students, or students' performance	Key findings	Interdisciplinary cooperation	DOI
18	**Stein SM et al. (2012) USA, study [10]**	Create template for presentations/lectures, course participants: College of Nursing educators, College of Pharmacy educators, panel: College of Education experts	One day teaching methods course including pedagogy, learning theory, teaching practice	Pre- and post-course video-recorded teaching presentations objectively assessed by expert panel, using validated evaluation tool. Participant pre- post-survey; objective results: significant improvement in 7 of 10 domains of teaching effectiveness	Yes - (undegraduate students may be impacted) - They raised awareness of the value of teaching effectiveness, thereby it may also support the scholarship of teaching.	1. anecdotal feedback: more effective teaching that year; 2. another participant created a poster for a national meeting on how the teaching methods course improved teaching effectiveness as measured through student surveys and subjective input	A short teaching-methods course can improve teaching effectiveness through enhanced communication and teaching. Training seminars can be integral to comprehensive quality improvement.	Yes	https://www.ajpe.org/content/ajpe/76/1/15.full.pdf
19	**Cole JD et al. (2019) USA, study [48]**	The study evaluated the effect of a university-affiliated pharmacy teaching certificate program on participant knowledge and perceived confidence in specific areas of academic pharmacy and preceptor development.	The 10-month program included 5 modules (Introduction to Teaching, Publications, Advanced Teaching Principles, Precepting, Graduation). Modules included writing a teaching philosophy statement, one-to-one mentoring, development on teaching pedagogy, assessment and evaluation.	Participant core-content knowledge was evaluated by written summative assessments (9-13 items of free response/multiple choice questions in 15 min.) pre-post modules. Test results were evaluated using paired t-tests. The online survey results showed significantly improved confidence rate to perform module objectives.	n/a	n/a	The lowest knowledge assessment scores were observed in the Introduction to Teaching (module 1) and Advanced Teaching Principles (module 3), these same modules also had the largest average difference in participant confidence.	n/a	10.1016/j.cptl.2019.07.002
20	**DiVall M et al. (2012) USA, study [49]**	The study focused on focus on teacher attitude to peer observation and evaluation. It aimed to evaluate a comprehensive, peer-driven teaching assessment program to promote improvement in teaching large enrollment classes.	The 4 steps of the intervention included: Pre-Observation Meeting, Classroom Observation, Post-Observation Meeting, and Post-Student Assessment Meeting. Thepeer-observation system was developed with the Center for Effective University Teaching. The program was mandatory for faculty formal teacher evaluation.	Pre-implementation survey assessed faculty needs and attitudes to peer program. 2-yr post-intervention, the repeated survey extended questions on faculty policy and perceived impact on teaching.	n/a	Student learning outcomes were not measured.	Faculty members had the most difficulty complying with step 4 (post-student assessment meeting), which was often absent.	Yes (with adult educational centre)	10.5688/ajpe76461
21	**Peters L et al. (2019) USA, study [23]**	The study utilized an online survey to detect and evaluate perceived gaps in skills, qualities, and knowledge needed for a successful career in academia. Survey aimed to identify topics to be included in the pharmacy residency training programs for better retention of qualified faculty educators.	The survey Ohio respondents (N=80) were former participants in residency teaching certificate programs (RTCP) of residency programs.	Perceived opinions of residency program participants from survey results.	n/a	no	Survey results showed hands-on pedagogical experience is most lacking from residency training programs. Faculty mentorship is needed and appreciated.	n/a	https://doi.org/10.1016/j.cptl.2019.07.012

No.	Authors, (date), country and publication category	Participants (population)	Pedagogy method used or referenced (intervention)	Evaluation of the results (outcome)	Are pharmacy students beneficiaries of the intervention, or will likely benefit?	Evaluation of effectiveness by undergraduate students, or students' performance	Key findings	Interdisciplinary cooperation	DOI
22	**Baia P and Strang AF (2016) USA, study [19]**	192 faculty and staff members employed at pharmacy schools with teaching roles before and after completing the HELP program - (2010-2014) survey - (online pedagogical professional development program)	Online professional development program titled Helping Educators Learn Pedagogy (HELP)	Qualitative and quantitative data analyzed for themes of motivation (data from written narratives, post-module quizzes, and survey) - data converted into units and coded	YES - INDIRECTLY - the HELP program includes developing lesson plans and assignments to increase student engagement and enhance student understanding	No - (but some positive student feedback reinforce long-term results)	Faculty educators must first value pedagogical knowledge for their continued growth as teachers, and the faculty development programs must appeal to value of learning, wish to improve student learning and to educators' beliefs regarding their roles and responsibilities.	n/a	10.5688/ajpe808132
23	**Lang B et al. (2019) China, study [15]**	The study presented a systematic examination of the effect of using Teat-Based Learning approach in pharmacy education in China. The 23 detected results referenced pedagogical issues and compared the effectiveness of team-based vs traditional lecture-based pedagogy based on test scores measuring student learning outcomes.	The pedagogical interventions showed minor variations depending on the specifics of the course where TBL was introduced. The interventions included educator responsibility of laying out the discussion topic, raising the questions (or cases), organizing post-tests and questionnaires, and evaluating effectiveness. The responsibility of student groups included preview, information search, literature reading, group discussion, and a summary report.	Scores of the objective theoretical test were considered as the primary outcome, and the results from questionnaires about the number of students who approved the effects of TBL pedagogy on improving their learning enthusiasm, self-study ability, thinking ability, and communication skills were considered as the secondary outcome.	yes	The measured results were based on student test scores and self-perceived student qualities.	Using Team-Based Learning pedagogy improved both theoretical test scores and self-perceived student qualities.	n/a	https://bmcmededuc.biomedcentral.com/articles/10.1186/s12909-019-1724-6
24	**Bradley F et al. (2011) UK, study [31]**	The study explored how hidden and informal curricula shaped student's learning about patient safety in two UK schools of pharmacy based on patient safety education as delivered. They examined patient safety content in the planned curriculum, also in the academic, organizational, and practice context.	The study draws on educational theory to organize the analysis into the categories of formal and informal curricula. Patient safety education as planned in the formal curriculum was analized based on documents and interviews with course or module leaders. The intervention methods included semi-structured interviews with participants and observations of teaching and clinical exposure sessions.	Eleven focus groups of study participants (N=44) included 2nd and 4th-yr MPharm students, Preregistration students, Newly Qualified Staff in hospital pharmacy and Clinical Pharmacy Educators. Qualitative results from audio recorded and transcribed interviews with participants were evaluated.	Informal exposure to practice is an important but sometimes hidden factor shaping the patient safety education of undergraduate pharmacy students. A major strength of the study lies in the project design, using a range of different data sources to examine the topic.	n/a	Educational courses may be transformed during delivery and implementation. Unofficial or informal social reality of a program needs investigating beyond the stated objectives or outcomes.	n/a	https://www.ajpe.org/content/75/7/143

No.	Authors, (date), country and publication category	Participants (population)	Pedagogy method used or referenced (intervention)	Evaluation of the results (outcome)	Are pharmacy students beneficiaries of the intervention, or will likely benefit?	Evaluation of effectiveness by undergraduate students, or students' performance	Key findings	Interdisciplinary cooperation	DOI
25	**Jasti BR et al. (2019) USA, study [50]**	The study described the development, implementation and assessment of a strategic plan and its process within a school of pharmacy. The 5-step plan included Designing and Scanning, Divergent Thinking, Convergent Planning, Refining, and Assessment. The inclusive approach ensured all stakeholders (faculty, staff, alumni, executive leadership,etc.) were represented and participated in the process.	Strategy 1 impacted curricular design and delivery by expanding active-learning methods across the curriculum, enhanced curricular mapping to ensure appropriate content coverage and sequencing, and enhanced communication among faculty members to enhance integration of content.	A general assessment was used based on metrics to evaluate the achievement of the objectives 3 years after implementation. The metrics were cross-referenced and aligned with the school assessment plan to ensure agreement and synergism. A mid-semester early warning system was introduced for at-risk students' benefit.	The results included a significant increase in students who participated in the presentation of research abstracts at professional and national scientific meetings. A midsemester early warning system had all faculty members provide lists of students at risk and then contact each student at risk with recommendations to use OASIS, advisors, instructors, and other resources.	The introduction of new career path advisors lead to a rise from 46% to 54% up to 71% in the percentage of graduating students which may indicate effectivity of guidance.	The overall strengths and uniqueness of the study include the use of a comprehensive approach, the establishment of a core strategic plan organizing committee (with 3 representatives from each academic department and an administrative staff member), the involvement of major stakeholders, and ongoing monitoring and assessment of the progress.	Yes (promotes linkage between the university's professional and liberal arts programs)	doi: 10.5688/ajpe6899
26	**Whitley HP et al. (2015) USA, review [24]**	The special article describes the benefits and barriers of Team-Based Learning (TBL), and the tools necessary for developing, implementing, and critically evaluating the technique within coursework in a user-friendly method. It uses a comprehensive approach by describing the general process of effective TBL development with detailed suggestions for the practical implementation of TBL in pharmacy education.	Suggestions for introduction of TBL include faculty workshops on TBL, peer mentoring or 'teaching circles' which provide peer-review, ensure quality improvement, and maintain consistency with course module. The primary instructor roles in TBL include promoting student-led discussions and teamwork, encouraging critical thinking, self-directed learning, monitoring group processes and progress, and creating learning environment.	The study recommends students and teachers should be pre-prepared for TBL to ensure feasibility and effectiveness of the program. Faculty performance evaluators must be able to differentiate student feedback on the pedagogy from feedback specific to a faculty member performance.	The study outlines the use of TBL accounts for improvement in student responsibility for own and group success. It suggested peer student evaluation scores can be used as a stand-alone component of the final course grade or used as a multiplier to "weight" team grades for each student.	The study suggests non-student evaluations (self- or peer faculty assessment) are advised to measure faculty performance, and student problem-solving skills are best to be tested at later stages.	Teachers need time to evolve as TBL facilitators; less obvious is the need for teacher support during declines in student and/or peer evaluations of teaching. Faculty members may require several years to improve, respond to peer feedback, and adapt teaching materials to TBL.	n/a	10.5688/ajpe7910149
27	**Lancaster JW et al. (2014) USA, study [9]**	The study classifies models of faculty development to make recommendations and collect best practices for the implementation of new programs. Based on the nine included publications, 3 primary models of faculty development were identified: centers, committees, and communities. It describes the benefits and disadvantagaes of the three program types.	Faculty learning community programs range from one-time events (eg, a retreat) to regular workshops or seminars, to highly competitive, application-driven, multi-month fellowship or scholar programs. Centers may offer grants, communication tools, assistance in collaborative reviews of teaching. Individual consultation has been recognized as one of the most effective faculty development practices.	The study highlights the key factors for determining a suitable faculty development model based on the nine result articles. In a review of teaching and learning centers, 3 general areas were assessed: satisfaction (eg, participation data, surveys); impact on teaching (eg, student evaluations, syllabus analysis, follow-up observation, focus groups), and impact on learning (eg, student retention, grade point averages, products of student learning).	n/a	"More research is necessary to maximize the outcomes of faculty development programs."	The main steps are: identify the area, target audience, and create a thorough and focused assessment plan. There is no best model due to the varying settings and needs.	n/a	https://doi.org/10.5688/ajpe78599

No.	Authors, (date), country and publication category	Participants (population)	Pedagogy method used or referenced (intervention)	Evaluation of the results (outcome)	Are pharmacy students beneficiaries of the intervention, or will likely benefit?	Evaluation of effectiveness by undergraduate students, or students' performance	Key findings	Interdisciplinary cooperation	DOI

28	**Law AV et al. (2012) USA, special article [51]**	The paper describes the faculty enrichment activities and outcomes of a faculty orientation and development committee using a quality improvement framework of needs assessment, planning and implementation of programs and workshops, assessment of activities, and evaluation of feedback in the College of Pharmacy of Western University of Health Sciences.	Developed programs were grouped in three categories: faculty orientation, faculty development and longitudinal faculty development. The faculty development workshops covered a broad range of topics including research methods, teaching (eg, test-item writing), and general development (mentorship). The luncheon workshops were scheduled for the same topic inviting internal or external speakers. The mentorship program had 15 pairs of participating educators.	Faculty surveys refected the committee's success by high levels of faculty attendance at workshops, positive feedback surveys on workshop evaluations, and the overall high levels of satisfaction with activities. Longitudinal faculty development involved formative peer assessment of teaching proficiency. Administrators (deans, department chairs, and directors) were yearly evaluated based on three categories: leadership, communication and management.	Students may be indirectly impacted by improved faculty proficiency, retention and improved atmoshere of work environment.	Students' course evaluations were included in the assessment component.	The stimulating and supportive work environment which facilitated good relationships increased job satisfaction and faculty retention. Faculty orientation offers a 'go-to' colleague as staff support for a new hires spanning for a one year to facilitate accommodation to the new environment and administration responsibilities.	n/a	https://doi.org/10.5688/ajpe7613
29	**Tweddell S et al. (2016) UK, study [25]**	The study aimed to assess faculty perceptions and experiences when implementing team-based learning (TBL) across a pharmacy curriculum. 40 % of respondents had no experience using other methods of teaching prior to using TBL.	The continuous collegial support during the intervention was beneficial through unscheduled informal meetings and sharing of personal experience. A qualitative method was selected to provide a deeper understanding of faculty perceptions, talking directly with small numbers of people sacrificing some scope for depth.	Academic staff participant survey results through semi-structured interviews were used to identify benefits and challenges of implementing TBL in the curriculum. Question areas covered Experience of Previous Teaching Methods, Initial Perceptions of TBL, Developmental Needs, Benefits, Challenges, Lessons Learned and Evolution of Practice.	The student-centered approach may require new pedagogic skills sets from educators. TBL requires an initial upfront investment in faculty development and time to prepare resources.	Did not measure student feedback.	The results showed the pedagogical benefits of engaging students in active learning, the development of transferable workplace skills, and the personal satisfaction after a TBL class outweigh the initial challenges.	Yes (national TBL expert cooperation initially and during in-house development program)	https://doi.org/10.1016/j.cptl.2015.09.008
30	**Smith L et al. (2017) USA, study [22]**	The study used an online survey to map common teaching activities in post-graduate residencies, identify residents' self-perceived teaching ability, and report characteristics of those feeling unprepared for an academic career by post-graduate residency training. (N=217)	The most common teaching activities participants felt prepared for included delivering didactic lectures, developing lecture handouts and exam questions, incorporating an active-learning exercise, writing a case study, providing student feedback and motivation.	The 16-item online survey was pretested and refined based on feedback provided by the institutions's Scholarship of Teaching and Learning (SoTL) Research Group.	No - (undegraduate students were not impacted)	no	Results found residents who felt unprepared for academia were exposed to significantly less teaching activities and reported less self-efficacy to perform the skills. The study underlines the results indicate that not only is the exposure to the teaching activity important but also the assessment method for mastery of the skill.	n/a	http://dx.doi.org/10.1016/j.cptl.2016.11.006
31	**Eksteen MJ et al. (2021) South Africa, study [26]**	The study examined whether TBL as a teaching strategy increases pharmacy students' understanding of theoretical work.	The intervention used team-based teaching strategy in a 4th yr pharmacy practice course. No information was provided on prior teacher pedagogical training. The intervention involved pre-class reading introducing new theory, its in-class application in professional context in student teams, and feedbacks.	Student survey (N=183) measured student agreement with the statements calculated in numerical results. The questionnaire contained biographical data and 16 items covering the following areas: Learning through Own Understanding, Learning from Others, Learning through Team-Based Learning and/or in Teams.	Students indicated that TBL not only promoted the achievement of learning outcomes set in the course, but also made it easier to achieve those outcomes.	Study results showed the undergraduate participants reported increased understanding of knowledge content, perceived higher knowledge retention, and better assessment results (compared with other parallel subject results).	Results showed setting the learning objective is primary, which constituted the selection of suitable pre-class reading. Another finding concluded learning from peers is enhanced by TBL, and prepares and improves working environment skills, such as interprofessional collaboration, or coping skills.	n/a	DOI: 10.7196/AJHPE.2021.v13i2.878
**No.**	**Authors, (date), country and publication category**	**Participants (population)**	**Pedagogy method used or referenced (intervention)**	**Evaluation of the results (outcome)**	**Are pharmacy students beneficiaries of the intervention, or will likely benefit?**	**Evaluation of effectiveness by undergraduate students, or students' performance**	**Key findings**	**Interdisciplinary cooperation**	**DOI**

32	**Rhoney DH et al. (2021) USA, study [21]**	The paper investigates the motivations and approaches to accelerate needed change in pharmacy education prompted by the demand for rapid restructuring in response to the COVID-19 pandemic. It presents a comprehensive outline of pharmacy education challenges, promotes ideation and provokes routes of change after the pandemic.	One referenced author suggested abandoning the obsolete viewpoint of departmental teaching, a shift in structuring the curricula as a complex adaptive network, and underlines to recognition of the need for increased collaboration among institutions.	The study describes academic, social, technological, economic, and political (ASTEP) forces driving the imperative to educational change.	n/a	not measured	The study authors argue it is time to disrupt current practices through innovation leading to new educational models and present sample solutions.	n/a	DOI: 10.1002/jac5.1407
33	**Wright EA et al. (2014) USA, journal article [52]**	The paper discussed a task force recommendations for the development and support of teaching and learning curriculum (TLC) experiences within postgraduate pharmacy training programs.	The policy statement and 12 recommendations to guide the development of best practices of TLC programs addressed topics including the value of TLC programs, program content, teaching and learning experiences, feedback for participants, the development of a teaching portfolio, the provision of adequate resources for TLC programs, programmatic assessment and improvement, program transparency, and accreditation.	The recommendations represent the concerted opinion of a professional task force which was reached based on input from the American Society of Health-System Pharmacists, reviewed evidence from literature, conference proceedings and considered author experience and expertise over a two-year period .	n/a	n/a	Postgraduate programs should be transparent to candidates and seek to ensure the best experiences for participants through systematic program implementation and assessments.	n/a	DOI: http://dx.doi.org/10.2146/ajhp130657
34	**Wolcott MD et al. (2023) USA, qualitative research study [53]**	The paper describes and evaluates how a design thinking (user-centered) approach aided the creation of the 2021 American Association of Colleges of Pharmacy (AACP) Teacher's Seminar.	An electronic survey was used to evaluate the perceived impact of using design thinking in the planning process. 9 people participated in the framework design using inspiration, ideation and implementation. 25 participants were recruited for the facilitator role.	After the seminar, the team was invited to debrief their experience in a focus group and an electronic survey to evaluate the perceived impact of using design thinking in the planning process.	The research focused exclusively on the designers and facilitators of the learning experience rather than the impact of the seminar participants.	no	Design thinking may be useful to help trainers connect with their anticipated audience, learn about their needs, generate options to pilot, prototype various solutions, and implement an option with continuous feedback systems.	n/a	https://doi.org/10.5688/ajpe8990
